# Investigating the Association between Sociodemographic Factors and Chronic Disease Risk in Adults Aged 50 and above in the Hungarian Population

**DOI:** 10.3390/healthcare11131940

**Published:** 2023-07-05

**Authors:** Amr Sayed Ghanem, Chau Minh Nguyen, Yara Mansour, Gergely Fábián, Anita Rusinné Fedor, Attila Nagy, Marianna Móré

**Affiliations:** 1 Department of Health Informatics, Institute of Health Informatics, Faculty of Health Sciences, University of Debrecen, 4028 Debrecen, Hungary; aghanem@etk.unideb.hu (A.S.G.); nguyen.chauminh@etk.unideb.hu (C.M.N.); yaramansour_@hotmail.com (Y.M.); 2 Institute of Social and Sociological Sciences, Faculty of Health Sciences, University of Debrecen, 4400 Nyíregyháza, Hungaryfedor.anita@etk.unideb.hu (A.R.F.)

**Keywords:** chronic diseases, cardiovascular disease, diabetes, sociodemographic factors, lifestyle factors, supplementation, heart disease, irritable bowel syndrome, cross-sectional study

## Abstract

Chronic diseases are a major cause of mortality and morbidity globally, with non-communicable diseases being responsible for most deaths. Older adults are at a higher risk of developing chronic diseases due to various sociodemographic and lifestyle factors such as age, sex, income, education, employment, place of residence, dietary supplementation, tobacco use, and alcohol consumption. Understanding the relationship between these factors and chronic diseases is crucial for identifying vulnerable populations and improving healthcare delivery. Through both an online and an interview-based survey, this cross-sectional study aimed to examine these associations, focusing on adults aged 50 and above, with the goal of identifying potential areas for intervention and prevention. The study found that gender, area of residence, education status, employment status, nutritional supplementation, body mass index (BMI), alcohol usage, and age are associated with the risk of chronic disease, cardiovascular disease, and diabetes. Female gender, higher educational level, employment, normal BMI, and younger age were found to be protective factors, while living in rural areas, alcohol consumption, and older age were identified as risk factors. The study recommends targeted interventions and improved access to healthcare to reduce risk factors and enhance healthcare delivery for better health outcomes.

## 1. Introduction

Chronic diseases, such as cardiovascular disease (CVD), stroke, diabetes, and cancer, are a leading cause of morbidity and mortality worldwide. Non-communicable diseases (NCDs) account for 71% of all deaths globally, and 85% of these deaths occur in low- and middle-income countries where chronic diseases are increasingly prevalent [1,2]. The burden of NCDs is expected to continue rising, particularly among older adults, as the global population ages and risk factors such as tobacco use, alcohol consumption, unhealthy diets, physical inactivity, and obesity become more common.

Sociodemographic factors, including age, sex, income, education, employment, and place of residence, have been shown to be associated with the development of chronic diseases [3]. Individuals aged 50 and above are more likely to develop chronic diseases and to experience disability and comorbidity than younger age groups. Sociodemographic factors are a wide array of different variables that could include older adults living alone, employment status, educational level, urban–rural living area. Understanding the associations between these factors and chronic diseases is essential for identifying populations at greater risk for these conditions, informing preventive interventions, and improving healthcare delivery.

Despite advances in medical technology and healthcare delivery, chronic diseases remain a significant challenge for healthcare systems worldwide. The burden of chronic diseases on individuals, families, and societies is substantial, with significant costs in terms of morbidity, mortality, and healthcare expenditure [4]. The prevalence of chronic diseases is expected to continue rising, particularly among older adults, as the global population ages. So, it is essential to understand the complex interplay of sociodemographic factors and chronic disease risk. Sociodemographic factors can influence access to healthcare, health behaviors, and the prevalence of risk factors for chronic diseases [5]. Previous studies have shown that sociodemographic factors affect chronic disease risk. However, the associations between these factors and chronic diseases in older adults still need more research to be properly explored and established [6].

Loneliness is increasingly recognized as a significant risk factor for a range of negative health outcomes, including cardiovascular diseases, health deterioration in diabetic patients, and mortality [7,8]. Older adults are at greater risk of experiencing loneliness due to a range of factors, including retirement, bereavement, and health problems. Employment status and educational level are also important predictors of chronic disease risk. Lower levels of education and unemployment have been associated with an increased risk of chronic disease and poorer health outcomes. Furthermore, urban–rural living areas may also be a factor, as rural areas may have limited access to healthcare resources and higher rates of poverty and chronic disease [9]. BMI, dietary supplementation, tobacco use, and alcohol consumption have also been linked to chronic disease risk in previous research [10,11].

Body mass index (BMI), a measure of body weight relative to height, is a strong predictor of chronic disease risk, particularly cardiovascular disease, and diabetes. A higher BMI is associated with a higher risk of these conditions [12]. Dietary supplementation, including the use of vitamins and other dietary supplements, has also been linked to chronic disease risk, while other supplements show potential protective effects [13]. Numerous studies have investigated the association between supplementary intake of calcium and vitamin C and the risk of cardiovascular disease, with some reporting an elevated risk [14,15]. Conversely, the scientific literature has consistently demonstrated the beneficial effects of vitamin D supplementation on cardiovascular health. Alcohol consumption is another factor that has been shown to influence chronic disease risk, with heavy drinking associated with an increased risk of several chronic conditions, including liver disease, cardiovascular disease, and cancer [11].

This study was done using data collected by the Faculty of Health Sciences, University of Debrecen, with the goal of identifying potential areas for intervention and prevention [16]. By understanding the associations between these sociodemographic factors and chronic disease risk, healthcare providers can work towards reducing the burden of these diseases and improving the health and well-being of older adults nationwide. The study’s results may have implications for public health policies and programs aimed at preventing and managing chronic diseases, particularly among vulnerable populations. The primary goal of this study was to identify any statistically significant correlations between these variables and the risk of developing chronic diseases over time in the target population of adults aged 50 years and older.

## 2. Materials and Method

The study employed a cross-sectional research design, set in the Hungarian city of Nyíregyháza. The data were collected in 2021.

### 2.1. Participants and Sampling

A priori sample size calculation was performed with a power level of 80% and an α level of 0.05 based on relevant literature research [17,18].

Hungarian adults aged 50 and above were allowed to participate in a survey that was conducted in two stages. The only inclusion criteria set was for the participants to be 50 years of age or older; data for participants younger than 50 years was not collected. The first stage happened in the first half of 2021, and consisted of an online, self-administered, questionnaire-based survey. The call to fill out the online survey was made available on the website and social media pages of the Faculty of Health Sciences, University of Debrecen. The online survey was also shared on private relevant social media groups after administrator approval, which contained members that exclusively represented the target age group and population. This approach to data collection at the first stage was selected due to multiple constraints that were placed on personal and social interactions due to the COVID-19 pandemic. The second stage of the survey was conducted by personally interviewing participants to help with the completion of the questionnaire. No specific interviewing technique was followed; the role of the interviewer was to provide help for the participants only. Sampling was done using group sampling after the COVID-19-related restrictions were lifted. Informed consent was provided at both stages of the data collection. Sampling and data collection were carried out by the “Health Awareness Workgroup,” affiliated with the University of Debrecen, Faculty of Health Sciences.

### 2.2. Study Methods

Data were collected using a questionnaire composed of questions from various validated questionnaires, which are referenced in the article [19,20,21,22]. The survey contained a total of 48 questions (Appendix A), divided into six categories. Each category contains a group of questions related to the topic of the given category. The first category was “Sociodemographic Information,” and it consisted of eight questions, including questions on age, gender, whether the participant lives alone or not, educational level, employment status, and area of residence, whether rural or urban. The second category was “Health Status,” consisting of 18 questions on the presence or absence of chronic conditions, and anthropometric measurements such as weight, height, and different health seeking behaviors. The third category was “Supplement Shopping Habits,” which focused mainly on nutritional supplements with four different questions. The fourth category was “Consumption Habits,” focusing on daily food and water consumption. The fifth category was “Health Literacy,” and the sixth category is “Attitude Towards Supplement Consumption,” focusing mainly on dietary probiotic supplementation. The focal point of this study was mainly the first two categories, which involve the sociodemographic characteristics and the health status of the participants.

### 2.3. Questionnaire Items

The focus area of this study was the association between different sociodemographic features of the study participants, and the presence of chronic diseases. Hence, from the six main categories of the study questionnaire, only data from the first two categories were used, namely “Sociodemographic information” and “Health Status”. These two categories contained a combined number of 26 questions. Under the “Sociodemographic information” category questions mainly focused on age, gender, whether the participant is living alone, or not. This specific question had five different answers, as the participants were able to report, if they do not live alone, with whom exactly do they live. To simplify the analysis and facilitate comparisons between living alone and living with others, the researchers collapsed the various responses to the question about living arrangements into two categories: “living alone” (i.e., those who selected “I live alone”) and “not living alone” (i.e., those who selected any of the other options). Participants were also asked to report their area of residence, with response options including “Rural”, “Small city”, “Large city” and “Municipality”. To facilitate analysis and simplify comparisons between rural and urban participants, the researchers collapsed the “Small city”, “Large city” and “Municipality” response options into a single “City” category. The educational background of the participants was assessed as well by asking them to report their highest educational degree obtained. Response options included “Less than 8 years elementary”, “8 years elementary”, “Vocational school degree”, “High school degree” and “University degree or higher”. To enhance comparability across participants with different levels of education, the researchers collapsed the “Less than 8 years elementary” and “8 years elementary” response options into a single “Elementary or less” category, and the “Vocational school degree” and “High school degree” response options into a single “High school or vocational” category. The employment status of the participants was assessed by asking them to report their current employment situation. Response options included “Employed,” “Unemployed,” “Pensioner,” and “Pensioner and employed.” To enhance the interpretability of the findings, the researchers collapsed the “Employed” and “Pensioner and employed” response options into a single “Employed” category.

Within the “Health Status” category, chronic disease was assessed using mainly two questions. The first question inquired about the presence of any chronic disease expected to last for six months or longer, with participants responding either “Yes” or “No”. This was a general question to allow the participants to self-report if they suffer from any kind of illness or health condition that lasts or is expected to last for at least 6 months, which includes but is not limited to cardiovascular disease and diabetes. This question was used as the basis for chi-squared tests to examine associations between sociodemographic factors and chronic disease risk, with significance threshold set at 0.05. The second question allowed participants to self-report whether they were currently suffering from a specific chronic disease such as cardiovascular disease, diabetes, peptic ulcer, or IBS, as well as other conditions such as allergies and high serum cholesterol. This was done to facilitate the analysis of the association between sociodemographic and lifestyle factors and chronic disease risk generally from one side and to analyze the same association with cardiovascular disease and diabetes alone.

### 2.4. Data Analysis

All categorical sociodemographic factors that exhibited significance in Pearson’s chi-squared test were incorporated into the regression models. For the multiple logistic regression models, the significant sociodemographic factors served as independent variables, while the dependent variables were chronic disease in general as well as cardiovascular disease and diabetes individually. These models identified the possible independent predictors of chronic disease, cardiovascular disease, and diabetes among the sociodemographic factors. Odds ratios (ORs) and the corresponding 95% confidence intervals [95% CI] were reported. Stata v17 (StataCorp. 2021. Stata Statistical Software: Release 17. StataCorp LLC, College Station, TX, USA) and Microsoft Excel 2018 were applied for data analysis.

## 3. Results

A total of 325 participants completed all survey questions at the first phase of the study, while those who did not were excluded. The second phase of the study consisted of personal interviews, resulting in 684 complete responses in total for the two phases combined.

Table 1 presents the sociodemographic characteristics of the study population, including the proportion of participants suffering from chronic diseases. Additionally, the table displays the prevalence of specific chronic diseases reported by the participants, including cardiovascular disease, diabetes, peptic ulcer, and irritable bowel syndrome. The table also shows proportion of the population who were smoking, and who were regularly drinking alcohol. The percentage of the study population regularly consuming vitamin and food supplementation was also displayed.

Out of the total 684 complete responses recorded, 42.5% were male and 57.5% were female. The age groups of participants were categorized as follows: 14% were above the age of 70, 30.85% were between the ages of 60 and 70 and 55.12% were between the ages of 50 and 59. In terms of living arrangements, 14.77% reported living alone while 85.23% reported living with others. Educational levels were classified into three categories: 8.48% had an elementary education or less, 46.63% had a high school or vocational education, and 44.88% had a university degree.

Regarding employment status, 2.49% reported being unemployed while below the age of retirement, 26.75% reported being pensioners, and 70.76% reported being employed. In terms of health behaviors, 67.54% reported consuming alcohol, 72.22% reported using supplement(s), and 31.87% reported being smokers. Body Mass Index (BMI) was categorized as either overweight or normal, with 25.73% of participants being overweight and 74.27% being of normal weight. In terms of residence, 70.03% lived in urban areas and 29.97% lived in rural areas.

Furthermore, Table 1 reports the prevalence rates of chronic diseases among the study population, including cardiovascular disease, diabetes, peptic ulcer, and irritable bowel syndrome (IBS). Overall, 32.6% reported having at least one chronic disease, with 41.23% reporting cardiovascular disease and 19.01% reporting diabetes. Only 4.68% reported having peptic ulcer and 4.53% reported having IBS.

The results presented in Table 2 were obtained using Pearson’s chi-squared test, with a significance threshold set at less than 0.05. The tables display the frequencies and percentages of various demographic and lifestyle factors stratified by the presence or absence of chronic disease. Significant differences between the two groups were observed for, education (*p* < 0.001), employment (*p* = 0.003), alcohol consumption (*p* = 0.006), supplements consumption (*p* < 0.001), BMI (*p* < 0.001), and gender categories (*p* < 0.001). However, no significant difference was found for residence (*p* = 0.06), living alone (*p* = 0.13), smoking (*p* = 0.054), and age categories (*p* = 0.088)

Table 3 shows the results of three separate binary logistic regression models. The dependent variables were chronic diseases, cardiovascular disease, and diabetes, respectively. The table displays the odds ratios with their corresponding 95% confidence intervals for the sociodemographic factors, including gender, area of residence, education status, employment status, nutritional supplementation, body mass index, living alone, alcohol usage, smoking, and age groups.

Gender was found to be a significant protective factor for chronic disease, with females having lower odds of developing chronic disease compared to males (OR 0.17, 95% CI [0.1–0.26]).

Area of residence showed a significant positive association with the development of cardiovascular disease, with individuals living in urban areas having higher odds of developing cardiovascular disease compared to those living in rural areas (1.53 [1.06–2.2]).

Education status was found to be a significant risk factor for chronic disease, with individuals who had only completed elementary or high school having higher odds of developing chronic disease compared to those who had completed university (2.22 [1.48–3.34]). The analysis used the proportion of the population with a university degree as a reference value, against which the odds were compared for other educational levels. The results showed that the odds were higher for individuals with only an elementary school degree or a high school degree, compared to those with a university degree. No significant association was found between education status and cardiovascular disease (0.81 [0.56–1.17]).

Employment status showed a significant protective association with chronic disease, with employed individuals having lower odds of developing chronic disease compared to those who were pensioners (0.49 [0.27–0.89]).

Nutritional supplementation showed a marginally significant positive association with cardiovascular disease in the univariate model, with individuals who reported using nutritional supplements having higher odds of developing cardiovascular disease compared to those who did not use supplements (1.33 [0.9–1.98]).

Body mass index (BMI) was found to be a significant protective factor for all three conditions. Individual within normal BMI range had lower odds of developing chronic disease (0.43 [0.28–0.66]), cardiovascular disease (0.43 [0.3–0.62]), and diabetes (0.36 [0.23–0.55]) compared to those with higher BMI.

Alcohol usage was significantly associated with cardiovascular disease (1.47 [1.02–2.11]) and diabetes (1.58 [1.01–2.46]), indicating that alcohol consumption could be a possible risk factor for these conditions.

The results also showed that smoking was not associated with the studied diseases.

Regarding age groups, individuals aged 50 to 59 years had a lower odds ratio for cardiovascular disease (0.28 [0.09–0.89]), and diabetes (0.54 [0.32–0.91]), compared to individuals aged 70 years or above. The same was apparent when the group of individuals aged 70 and above was compared to the other age group containing individuals aged between 60 and 70 years, having lower odds for cardiovascular disease (0.41 [0.21–0.80]) and diabetes (0.39 [0.17–0.85]).

## 4. Discussion

The present study aimed to investigate the prevalence of chronic diseases, CVD, and diabetes and identify their possible associations with sociodemographic and lifestyle factors, in a sample of adults aged 50 years or older. In this study, “chronic diseases” was utilized to collectively represent all chronic diseases including but not limited to CVD, diabetes, peptic ulcer, IBS, high serum cholesterol and other chronic long-standing conditions. However, separate analyses were also conducted for each category, such as examining cardiovascular disease in relation to various sociodemographic and lifestyle factors, and similar analysis was applied for diabetes. By conducting both collective and specific analyses, we aimed to gain a comprehensive understanding of the relationships between chronic diseases and various factors that may influence their occurrence. A study by Joel O Farnobi et al. (2020) suggested that several sociodemographic factors were significantly associated with these health outcomes, providing insights into potential risk and protective factors for these conditions [23].

This current study revealed that age was associated with both cardiovascular disease and diabetes. Specifically, individuals aged over 70 years exhibited higher odds of developing cardiovascular disease and diabetes compared to those aged 50 to 59 years. Two other studies by Jeffrey S. Bland et al. (2018) and Teresa Niccoli (2012) also found a similar link between age and chronic disease risk as aging is a known risk factor for many chronic diseases, including CVD and diabetes. However, this study adds to the literature by using a large sample size and a more diverse range of sociodemographic and lifestyle factors to better accentuate the relationship between aging and chronic disease risk in the Hungarian population [24,25].

Regarding gender, the results showed that females had lower odds of developing chronic diseases compared to males, which is in line with several previous studies done on the same topic [26,27]. However, no significant gender differences were observed when CVD and diabetes were analyzed separately, suggesting that the association between gender and these health outcomes may be complex and context specific. Biological factors such as hormones, genetics, and body composition may play a role. Additionally, social and environmental factors such as differences in occupational exposures, stress, and access to healthcare may also contribute to the gender gap in chronic disease risk. The lack of significance between gender, CVD and diabetes could also be explained by the fact that women lose the protective effect of the female hormones post menopause [28].

This study revealed that educational status was a significant factor associated with chronic diseases generally and diabetes, with individuals having higher educational levels having lower odds of developing these conditions. This finding is consistent with the original research paper by Collette Adamsen et al. (2018) that identified a link between educational status and chronic disease risk [6]. The protective effect of education against chronic diseases may be explained by several factors, including lifestyle behaviors, healthcare access, and other social determinants of health. The level of education has a direct effect on income, with people that have a higher level of education earning more on average than people with a lesser level of education. In the article mentioned above, it was established that high-income individuals are less likely to have a chronic disease in comparison to middle-to-low-income individuals. Moreover, this study adds to the existing literature by highlighting the relationship between educational status and chronic disease risk in the context of a specific geographic area, namely the city of Nyíregyháza, also providing a representative sample size with similar gender and urban/rural residence distribution to the Hungarian population. By examining this relationship in the above-mentioned population, this study provides important insights into the local factors that may influence the link between educational status and chronic disease risk.

According to the descriptive statistics regarding the level of education outlined in Table 1, more than 50% of the participants of this study did not have a university degree. So, encouraging the younger population to obtain a university degree could be a potential approach to lower the burden of chronic conditions in the future. Another approach could potentially be a targeted intervention for middle-to-low-income population and the marginalized communities to raise their level of education thus lowering their chronic illness risk. Implementation of policies that address the social determinants of health such as poverty, inequality, and wage rate could be a viable route to explore in this case, as cities in the eastern part of Hungary, where Nyíregyháza is also located, generally suffer from lower wage rates and lower levels of education in comparison to the capital city or the western part of the country.

Employment status was also found to be a significant factor associated with chronic diseases, with employed individuals having lower odds of developing the condition compared to unemployed individuals or pensioners. This indicates that employment status may have protective effects on health, potentially through the promotion of physical activity and social engagement. Regarding the previously cited article Collette Adamsen et al. (2018) a strong link was also found between employment status and the risk for chronic disease, suggesting that elderly who are employed exhibit a smaller likelihood of having chronic diseases in comparison to those who are either unemployed or pensioners [6]. Another article by Tazeen Majeed et al. (2014) where the same relationship was explored, the same conclusion was reached as in this article, as elderly who are employed generally exhibit better health and a lower chance of having chronic diseases [29]. The reason for this well-established association could be that employment provides individuals with a sense of purpose, social support, and a structured routine, which can promote healthier lifestyle behaviors such as regular physical activity and healthy eating habits. Additionally, employment can provide individuals with access to healthcare and resources that may be necessary to manage chronic conditions. On the other hand, unemployment and retirement may lead to social isolation, decreased physical activity, and financial stress, all of which can increase the risk of chronic diseases. In the present study, the previously established relationship between employment status and chronic diseases was confirmed and expanded upon by examining this relationship specifically in an older population above 50 years of age in a Hungarian city. These findings contribute to the growing body of evidence highlighting the importance of employment as a protective factor against chronic diseases and suggest that promoting employment opportunities among older individuals may have significant health benefits. Additionally, this study adds to the limited literature on this topic in Eastern Europe, providing valuable insights for policymakers and healthcare professionals in the region. Providing part-time jobs for pensioners could be a flexible job opportunity that gives them a sense of purpose and adds to their limited pension income. Pensioners could also serve as mentors or coaches to younger employees in their field. Volunteer work could also be considered providing the elderly with an opportunity to help others, and a chance to socially connect, reducing the effects of loneliness and isolation.

Interestingly, alcohol usage was found to be a risk factor for the CVD and diabetes subcategories, but when association was measured with the general category of chronic diseases, no statistical significance was found. This just underlines what is already known from existing scientific literature, which has suggested that heavy alcohol consumption may increase the risk of heart disease and diabetes. However, the relationship between alcohol and chronic diseases may also depend on factors such as the type and amount of alcohol consumed, and this was not included in the scope of this study. Participants tend to underestimate their alcohol consumption and tend to underreport it, fearing the judgment of others, so this is a main limitation for self-reporting alcohol consumption. As a consensus though, heavy drinking is a potential aggravating factor in the risk of CVD and diabetes, and this effect only increases when confounded by older age [11]. The study adds to the existing body of literature on the association between alcohol consumption and chronic diseases, particularly cardiovascular disease, and diabetes. Specifically, it provides evidence on the prevalence of alcohol consumption in a specific population in Eastern Europe, as well as its relationship with chronic disease subcategories. This information can be used to inform future research and interventions aimed at reducing the burden of chronic diseases in this population and beyond. Additionally, the study’s findings on the potential moderating factors of the alcohol–chronic disease relationship contribute to a deeper understanding of the complexities involved in this association.

Approximately 26% of the total participants were classified as overweight based on their BMI. Subsequent statistical analyses revealed that being overweight was associated with significantly increased odds of having chronic diseases, cardiovascular disease, and diabetes. These findings highlight the significant role of excess weight as a risk factor for multiple chronic diseases, including cardiovascular disease and diabetes. Another study by Syed Afroz Keramat et al. (2021) also concluded that excessive weight in Australian middle-aged and older adults is significantly correlated with a higher incidence of chronic disease [30]. The mechanism underlying the association between overweight and chronic diseases is multifaceted and may be attributed to a range of factors, including insulin resistance, chronic inflammation, dyslipidemia, and endothelial dysfunction. An original article researching the effects of obesity on chronic disease in Ireland stated that even a small reduction in the average BMI of the general population has a significant impact on the burden of chronic disease [12]. The current study reinforces the importance of weight management, adding to the preexisting literature by exploring this relationship in the elderly population in Hungary along with the effects of other variables that could confound this relationship, such as the level of education and the area of residence, and it also highlights the need for targeted interventions aimed at reducing the prevalence of excess weight in the older adult and elderly population, who are already at an increased risk of having chronic conditions. This could involve implementing community-based programs that promote healthy eating and physical activity, as well as increasing access to resources that support weight management, such as nutrition counseling and exercise programs. In addition, the study emphasizes the importance of regular health screenings, especially for older adults, in order to identify those at risk for chronic diseases and provide early intervention and management. It was also suggested that chronic conditions related to increased weight are not mediated solely by overall body fat mass; the distribution of the body fat also plays a significant role in mitigating or accentuating the effects of this increased adipose mass. For example, metabolic syndrome mainly appears in men, with the main fat mass concentrated along the waistline, resulting in what is called the “apple shaped” body type [31]. Thus, future research should also include a waist circumference measure besides the BMI so that this relationship can be explored on a deeper level.

Most participants who reported not living alone stated living with their spouses (74.6%), while 24.7% reported living with their children. Only 14.8% of the total participants reported living alone. Surprisingly, no significant association was found between living alone and the risk of chronic disease in this study. This finding is somewhat unexpected, as previous research has suggested that living alone is a risk factor for chronic diseases in the elderly population. Citing other research, it was concluded that accidents such as injuries due to falls and other pathologies such as vision loss and hearing loss are attributed to the elderly living alone. Aggravating already present conditions of chronic illness were also linked to the same reason due to the difficult mobility of older adults, especially the elderly, resulting in reduced follow-up with the district physician [32,33]. It would be interesting to explore this topic further in future research in the same eastern European region where research on this topic was scarcely conducted. Future research may need to consider using larger and more diverse samples and employing longitudinal designs to examine the relationship between living alone and chronic disease risk over time.

The study also showed a significantly higher prevalence of diabetes (19%) among the study participants compared to the reported national average of 9.1% by the International Diabetes Federation [34,35]. There are several potential reasons for this discrepancy. It is important to note that the participants in this study were older adults and elderly individuals, who may be at higher risk for developing diabetes compared to the general population. In fact, previous research has suggested that the prevalence of diabetes increases with age [36]. Therefore, it is possible that the higher proportion of diabetes in this study is reflective of the age distribution of the participants.

A marginally positive association was found between the use of vitamin and nutritional supplementation and the development of cardiovascular disease. It is plausible that this result is attributable to the types of supplements used, particularly given that many older adults and elderly individuals in this study reported using vitamin and nutritional supplements, the proportion of participants using these products was as high as 72%. For example, a large portion of the given target population use calcium supplementation for osteoporosis prevention. In addition, the COVID-19 pandemic led to increased marketing and consumption of vitamin C supplements among older adults and elderly individuals as a means of strengthening the immune system against infections, given that COVID-19 posed an elevated risk to this demographic. Studies have shown that both supplemental calcium and vitamin C increase the risk of heart disease, which may explain the observed association in this study [14,15]. Nevertheless, further research is required in this area to draw definitive conclusions. The implications of these findings for public health suggest that older adults and elderly individuals should exercise caution when using supplements and consider consulting with a healthcare professional before taking any new supplements. Furthermore, these findings highlight the importance of continued research in this area to identify the specific types of supplements and dosages that may pose risks for cardiovascular disease and other chronic diseases.

The observation that area of residence was significantly associated with cardiovascular disease risk in this study is in harmony with what was previously concluded in scientific literature [37,38]. Prior research has already established that citizens of rural areas generally have poorer health, larger waist circumference, less physical activity, higher total cholesterol levels and worse dietary patterns than urban populations [32]. Another reason for this discrepancy is the difference in access to healthcare and medical services between rural and urban areas. Rural areas in eastern Hungary are often characterized by a shortage of medical professionals, limited access to healthcare facilities, and longer travel distances to reach medical services. These factors may result in delayed diagnosis and treatment of cardiovascular disease, leading to worse health outcomes in individuals residing in rural areas [39].

Although smoking is a well-known risk factor for chronic disease, cardiovascular disease, and diabetes in the scientific literature, this study did not find a significant association between smoking and any of the three outcomes [40]. It is noteworthy that 68% of the participants reported being non-smokers, which is in perfect line with research done on the prevalence of tobacco use in Hungary which stated that the prevalence of smoking in the country is around 31% [41]. This adds to the evidence that the current study does indeed represent the larger Hungarian population from multiple aspects. One reason though, to explain this result could be that the participants who smoked may have quit smoking before the study, thus reducing their risk of chronic diseases. Also, participants tend to underreport and underestimate their smoking status. Additionally, there could be other factors that contribute to the development of chronic diseases, such as physical activity level, genetic predisposition to certain chronic conditions, diet, and environmental factors, which may have confounded the relationship between smoking and chronic diseases. However, it is important to note that the lack of a significant association between smoking and chronic diseases in this study does not mean that smoking is not a risk factor. It is still critical to encourage smoking cessation and prevent smoking initiation to reduce the burden of chronic diseases in the population. Further research with a larger sample size and a more diverse population is necessary to confirm the relationship between smoking and chronic diseases in this population.

The global prevalence of irritable bowel syndrome is estimated to be approximately 11%, with previous studies in the Hungarian population indicating a prevalence range of 10–15% [42,43,44]. However, in this study, the prevalence of IBS was found to be less than 5%, which is notably lower than expected. Further research is necessary to elucidate the reasons underlying the low IBS prevalence in this particular population. This very low prevalence was the reason for both IBS and peptic ulcer patients to be excluded from the multivariate model, as their very small sample size will not provide enough statistical power to explore the relationship between them and other variables.

### Strengths and Limitations

It was observed that the survey had a representative distribution of gender among the participants. Additionally, the study showed a balanced representation of area of residence, reflecting the population of Hungary in terms of rural and urban areas. The small sample size of individuals living alone may have limited the power to explore the relationship between individuals living alone having a higher risk for chronic diseases. The study also reported a higher proportion of diabetic participants than the national average. Differences in diagnostic criteria and measurement methods may have contributed to this higher proportion. Also due to the cross-sectional nature of this study, individuals self-reported their diagnosis which may be subject to recall bias thus not accurately reflecting the true prevalence of this condition.

There was a lack of statistical association between smoking and the risk for chronic diseases, CVD and diabetes which could be down to misclassification of the smoking status, or selection bias or the relationship between smoking and chronic conditions could be confounded by other variables.

## 5. Conclusions

The study offers significant insights into sociodemographic and lifestyle variables associated with chronic diseases in individuals over 50 years old. These findings have practical implications for targeted interventions and improvements in healthcare. Healthcare professionals can develop focused treatments and interventions that address specific risk factors, such as promoting healthy diets, increasing physical activity, and reducing alcohol intake. Additionally, efforts should be made to enhance access to healthcare, particularly in rural regions, to facilitate early recognition and management of chronic disease risk factors.

Encouraging healthy lifestyle practices through counseling, health education campaigns, and community outreach programs is crucial, given the impact of lifestyle variables on chronic disease risk. Health education programs, especially tailored to older populations, can raise awareness about the importance of healthy living and chronic disease prevention. Moreover, improving the level of education can play a significant role in promoting healthy behaviors and reducing the likelihood of developing chronic illnesses.

Lastly, healthcare practitioners should implement age-appropriate screening and preventive approaches to detect and manage chronic disease risk factors in older populations. By recognizing and addressing these risk factors early, healthcare professionals can contribute to a decrease in the prevalence of chronic illnesses in older populations.

In conclusion, the study findings guide focused initiatives to lower the prevalence of chronic diseases, enhance healthcare delivery, raise public understanding of the value of healthy lifestyle choices in avoiding chronic diseases, and improve the level of education to promote healthier behaviors. Implementing these advancements can support improved health outcomes for senior citizens and lessen the burden of chronic illnesses nationwide.

## Figures and Tables

**Table 1:** Sociodemographic characteristics of the participants, proportions of chronic diseases (N = 684).

	N	%
**Gender**		
Male	290	42.50%
Female	394	57.50%
**Age groups**		
Above 70	96	14.04%
60 to 70	211	30.85%
50 to 59	377	55.12%
**Living alone**		
Yes	101	14.77%
No	583	85.23%
**Education**		
Elementary or less	58	8.48%
High school or Vocational	319	46.63%
University degree	307	44.88%
**Employment**		
Unemployed	17	2.49%
Pensioner	183	26.75%
Employed	484	70.76%
**Alcohol consumption**		
User	462	67.54%
Non-user	222	32.46%
**Supplement consumption**		
Yes	494	72.22%
No	190	27.78%
**Smoking**		
Smoker	218	31.87%
Non-Smoker	466	68.13%
**BMI**		
Overweight	176	25.73%
Normal	508	74.27%
**Residence**		
City	479	70.03%
Rural	205	29.97%
**Chronic Disease**		
Yes	223	32.6%
No	461	67.4%
**Cardiovascular Disease**		
Yes	282	41.23%
No	402	58.77%
**Diabetes**		
Yes	130	19.01%
No	554	80.99%
**Peptic Ulcer**		
Yes	32	4.68%
No	652	95.32%
**IBS**		
Yes	31	4.53%
No	653	95.47%

**Table 2:** Association of chronic disease risk with different socioeconomic and lifestyle factors.

	Chronic Disease		
	Yes (n = 223)	No (n = 461)	*p*-Value
**Residence (n%)**			
City	167 (34.9%)	312 (65.1%)	0.060
Rural	56 (27.3%)	149 (72.7%)	
**Living alone (n%)**			
Yes	26 (25.7%)	75 (74.3%)	0.130
No	197 (33.8%)	386 (66.2%)	
**Education (n%)**			
Elementary or less	7 (12.1%)	51 (87.9%)	**<0.001**
High school or vocational	75 (23.5%)	244 (76.5%)	
University degree	141 (45.9%)	166 (54.1%)	
**Employment (n%)**			
Unemployed	5 (29.4%)	12 (70.6%)	**0.003**
Pensioner	78 (42.6%)	105 (57.4%)	
Employed	140 (28.9%)	344 (71.1%)	
**Alcohol consumption (n%)**			
User	139 (30.1%)	323 (69.9%)	**0.006**
Non-User	84 (37.8%)	138 (62.2%)	
**Supplement consumption (n%)**			
Yes	190 (38.5%)	304 (61.5%)	**<0.001**
No	33 (17.4%)	157 (82.6%)	
**BMI (n%)**			
Overweight	76 (43.2%)	100 (56.8%)	**<0.001**
Normal	147 (28.9%)	361 (71.1%)	
**Gender**			
Male	147 (50.5%)	143 (49.5%)	**<0.001**
Female	49 (12.4%)	345 (87.6%)	
**Smoking (n%)**			
Smoker	60 (27.5%)	158 (72.5%)	0.054
Non-Smoker	163 (35%)	303 (65%)	
**Age groups (n%)**			
70 years of age or above	33 (34.4%)	63 (65.6%)	0.088
Between 60 and 70	80 (37.9%)	131 (62.1%)	
50 to 59	110 (29.2%)	267 (70.8%)	

Bold values indicate statistical significance (*p* < 0.05) based on chi-squared tests.

**Table 3:** Association of different sociodemographic and lifestyle factors with chronic disease, cardiovascular disease and diabetes risk.

Sociodemographic Factors	Chronic Diseases	CVD	Diabetes
	(OR [95% CI])	(OR [95% CI])	(OR [95% CI])
**Gender**			
Female/Male	**0.17 [0.1–0.26]**	0.73 [0.5–1.07]	0.71 [0.44–1.15]
**Area of residence**			
Rural/City	0.88 [0.58–1.34]	**1.53 [1.06–2.2]**	1.11 [0.7–1.75]
**Educational status**			
Elementary/University	**7.49 [2.86–19.58]**	0.81 [0.56–1.17]	0.66 [0.30–1.47]
High School/University	**2.22 [1.48–3.34]**	0.83 [0.43–1.61]	**0.52 [0.33–0.83]**
**Employment status**			
Unemployed/Employed	0.61 [0.17–2.13]	0.82 [0.5–1.37]	0.85 [0.47–1.53]
Pensioner/Employed	**0.49 [0.27–0.89]**	0.94 [0.33–2.69]	0.82 [0.46–1.48]
**Nutritional supplementation**			
Yes/No	0.67 [0.41–1.11]	1.33 [0.9–1.98]	0.74 [0.44–1.23]
**Body Mass Index**			
Normal/Overweight	**0.43 [0.28–0.66]**	**0.43 [0.3–0.62]**	**0.36 [0.23–0.55]**
**Living alone**			
Yes/No	1.44 [0.82–2.52]	0.98 [0.62–1.55]	0.95 [0.54–1.67]
**Alcohol usage**			
User/Non-user	1.16 [0.77–1.74]	**1.47 [1.02–2.11]**	**1.58 [1.01–2.46]**
**Smoking**			
Smoker/Non-smoker	0.9 [0.58–1.39]	1.22 [0.83–1.78]	0.95 [0.59–1.52]
**Age groups**			
50 to 59/70 and above	0.71 [0.19–2.68]	**0.28 [0.09–0.89]**	**0.54 [0.32–0.91]**
≥60 but <70/70 and above	0.67 [0.30–1.48]	**0.41 [0.21–0.80]**	**0.39 [0.17–0.85]**

OR: odds ratio, 95% CI: 95% confidence interval. Bold values represent significant association.

## Data Availability

The data are available upon request.

## Appendix A. Questionnaire

SOCIODEMOGRAPHICS

1. What is your gender?
  1. Female
  2. Male

2. How old are you?
  ..........years

3. Who do you live with?
  1. with my spouse
  2. alone
  3. with my child
  4. with my grandchild
  5. other: ...................

4. Who visits you regularly?
  1. my child/children
  2. other family members
  3. friends, acquaintances
  4. neighbours
  5. social helpers
  6. no one visits me regularly
  7. other: ...................

5. What type of settlement do you live in?
  1. village
  2. small town
  3. big city
  4. city with county rights

6. What is your highest level of education?
  1. less than 8 primary school years
  2. 8 primary school years
  3. Vocational school training
  4. High school
  5. University

7. What is your labour market situation?
  1. I work
  2. I am unemployed
  3. I am retired, but I work alongside
  4. I am retired and no longer work

8. How do you assess your financial situation?
  1. very good
  2. good
  3. average
  4. bad
  5. very bad

HEALTH STATUS

9. How would you describe your health in general?
  1. Very good
  2. Good
  3. Average
  4. Bad
  5. Very bad

10. How much do you think you can do for your health?
  1. I can do a lot of things
  2. I can do a few things
  3. there is little I can do
  4. there is nothing I can do

11. To what extent do you think the consumption of food supplements can affect your health?
  1. to a very large extent
  2. to a large extent
  3. to an average extent
  4. to a small extent, barely
  5. not at all

12. Do you suffer from a chronic (long-standing, slow-onset) illness that has lasted or is expected to last for at least 6 months?
  1. Yes
  2. No

13. In the last 12 MONTHS, have you had any illness or health problem from the list?
  1. Heart and vascular disease or related conditions (heart attack; coronary heart disease, angina; high blood pressure; stroke; arrhythmia, other heart disease)
    Yes/No
  2. Diabetes/Diabetes
    Yes/No
  3. Stomach or duodenal ulcer
    Yes/No
  4. IBS (irritable bowel syndrome)
    Yes/No
  5. Allergy
    Yes/No
  6. High cholesterol, fat metabolism disorder
    Yes/No
  7. Other: ..................................................

14. In the last 12 MONTHS, how many times in the last 12 MONTHS have you seen/contacted your GP by phone for your own health?
  .......... times

15. In the last 12 MONTHS, how many times have you met/contacted a specialist doctor on the phone about your health?
  .......... times

16. In the last 12 MONTHS, have you ever needed to see a specialist but not done so?
  1. yes, at least once
  2. no

17. What types of complaints do you usually consult a doctor about? Please name the most common reason.
  1. Even if you are not ill or have no complaints—you like to know how your health is.
  2. Even with minor aches and pains.
  3. With more pain, more complaints
  4. Only if you are in a lot of pain.
  5. If someone close to you will take you to the doctor, otherwise you wouldn’t go.
  6. Don’t know

18. Do you know how to manage diabetes?
  Yes
  No

19. Do you know what complications diabetes can have?
  Yes
  No

20. If you have diabetes, have you had any complications?
  Yes
  No

21. If you have had complications, do they limit your daily activities?
  1. yes, to a very large extent
  2. to a large extent
  3. to an average extent
  4. to a small extent, barely
  5. not at all

22. How tall are you without shoes? ………..cm

23. What is your weight without clothes and shoes? ……… kg

24. Do you smoke even occasionally?
  1. yes, daily
  2. yes, a few times a week
  3. yes, once a week (on weekends)
  4. yes, but even less often
  5. no, never
  6. I used to smoke, but I quit

25. Do you drink alcohol even occasionally?
  1. yes, daily
  2. yes, a few times a week
  3. yes, once a week (on weekends)
  4. yes, but even less often
  5. no, never
  6. I used to consume it, but no longer do

26. How often do you have a bowel movement?
  1. Once a day
  2. Twice a day
  3. More than two times a day
  4. Irregularly
  5. I am constipated all the time

SHOPPING HABITS

27. Do you buy vitamin/nutritional supplements?
  1. yes
  2. not

28. What are the main factors that determine which vitamin/nutritional supplements you choose? (you can choose 3)
  1. the price of the product
  2. the manufacturer of the product
  3. the advertisements
  4. what I heard about it
  5. what my previous experiences have been
  6. what disease I suffer from
  7. other:

29. Whose opinion matters to you when choosing a vitamin/nutritional supplement?
  1. my partner’s
  2. my child’s
  3. my grandson’s
  4. the supplement shop salesperson
  5. a specialist (e.g., GP, specialist, pharmacist)
  6. other: …..

30. Do you read the product descriptions, ingredient guides?
  1. Yes, always
  2. Sometimes
  3. Never

NUTRITIONAL CHARACTERISTICS

The following questions concern your habits.

31. How much fluid do you drink per day?
  1. less than 1 litre
  2. 1–2 litres
  3. 2–3 litres
  4. more than 3 litres

32. Which meal do you include in your daily eating routine?
  1. breakfast
  2. brunch
  3. lunch
  4. afternoon snack
  5. dinner

33. Do you take a vitamin/supplement regularly?
  Yes
  No

34. If you are taking these products, at what time of day do you take them?
  Morning
  At noon
  Evening

35. For you, what would be the most appropriate vitamin/nutrient supplementation routine?
  Once a day
  Twice a day
  Three times a day

HEALTH LITERACY

Please tick how often the following statements are true for you! Next to each of them, you will find a number line from 0 to 4, indicating the degree to which the statement applies to you. When filling in the form, please note that there are no right or wrong answers, the aim is to get an accurate picture of your daily orientation in the field of health and food related publications guides!
  0—Never   1—Seldom   2—Sometimes   3—Most of the time   4—Always

36. How often can you interpret product labels and instructions independently?
  0 1 2 3 4

37. How often does someone (e.g., family member, friend, acquaintance, salesperson, carer) help you interpret product descriptions?
  0 1 2 3 4

38. Do you have problems understanding the instructions, getting a good picture of the product, whether you can consume it?
  0 1 2 3 4

CONSUMER ATTITUDES SURVEY

39. Have you ever heard the term probiotic?
  (a) yes   (b) no   (c) do not know

40. If you have heard the term before, where did you get the information?
  (a) doctor   (b) pharmacist   (c) family member/acquaintance
  (d) newspaper/leaflet   (e) advertisement   (f) internet   (g) don’t know

41. Which statements do you think describe probiotics?
  (a) have a beneficial effect on intestinal flora
  (b) living micro-organisms
  (c) contain beneficial bacteria
  (d) a type of yoghurt preparation
  (e) immune-boosting preparation

42. Have you ever bought a probiotic?
  (a) yes   (b) no   (c) don’t know/no answer

43. If you have not yet purchased a probiotic, why would you do so? (you can tick more than one answer)
  (a) I am healthy, I do not need it
  (b) such a product must be very expensive
  (c) I don’t know if such a product would be useful for me
  (d) unnecessary waste of money because I eat healthy
  (e) I consider such products as a fashion fad

44. For what purpose did/would you buy probiotics?
  (a) together with a course of antibiotics
  (b) independently of a course of antibiotics, as an additional treatment for a disease
  (c) for the prevention of disease

45. If you bought probiotics, what factors played a role in your purchase? Rank the statements 1 to 7 (most important 1, least important 7)
  I tried it and it has a good effect on me
  I look for the product according to my disease
  the price of the product
  accurate knowledge of the health benefits of the product
  a friend/family member has tried it
  I am buying the product for preventive purposes
  I know about new products from advertising

46. What factors do you think would help you buy vitamins/nutritional supplements/probiotics? (you can tick more than one answer)
  (a) if it were indicated on the product for which disease it should be consumed
  (b) if it were indicated on the product which disease it could prevent
  (c) if the product were labelled with an indication of the age group for which it should be purchased
  (d) if the product would contain any herbs

47. What form of vitamin/supplement/probiotic would you prefer to take?
  (a) capsules
  (b) in liquid form
  (c) in the form of liquid soluble powder

48. Do you take more vitamins/nutritional supplements/probiotics as you get older?
  (a) no, because it is a waste of money to spend on them
  (b) no, because I get all the vitamins and minerals I need from my diet
  (c) no, because my health does not require it
  (d) yes, because I need to be more aware of myself as I get older
  (e) yes, because my health changed, I became ill
  (f) yes, because foods contain less and less good/useful nutrients
  (g) I do not know
